# Predicting Soil Organic Matter, Available Nitrogen, Available Phosphorus and Available Potassium in a Black Soil Using a Nearby Hyperspectral Sensor System

**DOI:** 10.3390/s24092784

**Published:** 2024-04-27

**Authors:** Shuming Wan, Jiaqi Hou, Jiangsan Zhao, Nicholas Clarke, Corné Kempenaar, Xueli Chen

**Affiliations:** 1 Heilongjiang Academy of Black Soil Conservation and Utilization, Heilongjiang Academy of Agricultural Sciences, Harbin 150086, China; shuming.wan@haas.cn; 2 Agrosystems Research, Wageningen University & Research, P.O. Box 16, 6700 AA Wageningen, The Netherlands; 3 State Key Laboratory of Environmental Criteria and Risk Assessment, Chinese Research Academy of Environmental Sciences, Beijing 100012, China; 4 Norwegian Institute of Bioeconomy Research, P.O. Box 115, N-1431 Aas, Norway

**Keywords:** hyperspectral sensor, black soil, soil moisture, partial least squares regression, feature wavelength

## Abstract

Black soils, which play an important role in agricultural production and food security, are well known for their relatively high content of soil organic matter (SOM). SOM has a significant impact on the sustainability of farmland and provides nutrients for plants. Hyperspectral imaging (HSI) in the visible and near-infrared region has shown the potential to detect soil nutrient levels in the laboratory. However, using portable spectrometers directly in the field remains challenging due to variations in soil moisture (SM). The current study used spectral data captured by a handheld spectrometer outdoors to predict SOM, available nitrogen (AN), available phosphorus (AP) and available potassium (AK) with different SM levels. Partial least squares regression (PLSR) models were established to compare the predictive performance of air-dried soil samples with SMs around 20%, 30% and 40%. The results showed that the model established using dry sample data had the best performance (RMSE = 4.47 g/kg) for the prediction of SOM, followed by AN (RMSE = 20.92 mg/kg) and AK (RMSE = 22.67 mg/kg). The AP was better predicted by the model based on 30% SM (RMSE = 8.04 mg/kg). In general, model performance deteriorated with an increase in SM, except for the case of AP. Feature wavelengths for predicting four kinds of soil properties were recommended based on variable importance in the projection (VIP), which offered useful guidance for the development of portable hyperspectral sensors based on discrete wavebands to reduce cost and save time for on-site data collection.

## 1. Introduction

Black soils (Mollisols in the USDA soil taxonomy) are one of the most important soil resources in the northeast of China [1] and play an important role in maintaining food security [2]. Soil organic matter (SOM) is a central component of the terrestrial carbon cycle [3], with a significant impact on the sustainability of farmland [4], for example, providing a primary source of nutrients for plants, helping to aggregate particles and develop soil structure, increasing water storage capacity and availability for plants, protecting soil from eroding and providing a habitat for soil biota [5]. In order to manage soil nutrients in an efficient way and fertilize crops according to their needs, it is necessary to obtain detailed information about farmland soil properties, for example, SOM, available nitrogen (AN), available phosphorus (AP) and available potassium (AK) [6].

Soil sampling protocols and conventional laboratory analyses can be used to measure soil properties. The methods are accurate but time-consuming and labor-intensive because a large number of samples is required to capture spatiotemporal variability [5]. Assessment of soil properties across time and space, as well as a fast and accurate method for quantification of nutrients, is important for sustainable agricultural and environmental management [7]. However, rapid and reliable assessment of soil characteristics has become one of the great challenges in soil monitoring.

Hyperspectral imaging (HSI) is a non-destructive method that can provide detailed and highly resolved reflectance characteristics of target materials on different scales, and it has the advantage of capturing both spatial and spectral information [8]. Currently, spectrometry in the laboratory has been widely applied to the quantitative inversion of SOM based on the organic matter-sensitive bands that exist in the visible range of 550–770 nm and the near-infrared range of 1300–1500 nm [4,9]. Yu et al. [10] found that the correlation coefficients of soil organic carbon with the bands in the near-infrared wavelengths of 747 to 1000 nm and 1010 to 1136 nm were significant at the significance level of 0.01. For similar near-infrared wavelengths, the correlations of total phosphorus, total nitrogen and total potassium were significant at the significance level of 0.05. Yang, Bao, Li, Liu, Fu and Mao [9] found that unmanned aerial vehicle (UAV) HSI can be exploited to quantify SOM and soil total nitrogen in farmland. This technique was therefore successfully applied to evaluate the spatial variability of SOM and other soil properties within both small profiles and on a large scale. 

Soil moisture (SM) is an important parameter in crop growth and produces a significant variation in soil spectral reflectance [11,12]. For wet soils, the difference between the refractive index of soil (nsoil, λ approximately 1.50) and water (nwater, λ approximately 1.33) is smaller than that of dry soils, where the particles are surrounded by air (nair, λ approximately 1.00). This decrease in refractive index at the soil–water–air surfaces produces a lower scattering of incident light [13]. Moreover, the SM will influence the prediction of soil properties due to the inherent correlation between water and spectrally active soil components, for example, SOM and AN [14]. The related overtones of O-H stretching and combinations of H-O-H bending and O-H stretching conceal the absorption of soil N-H in the near-infrared (NIR) spectroscopy, resulting in the spectral curve only reflecting the changing trend of soil moisture [15,16]. While in the laboratory, soil can be scanned under standard air-dried conditions, but in the field, it is very difficult to control the water content. Variation in soil moisture can mask, at varying degrees, the spectral response of soil properties, causing a decrease in prediction accuracy. Morgan et al. [17] showed that when using NIR on field samples, the variability of soil moisture in the field reduced the prediction accuracies of soil organic carbon (SOC) content. However, additional algorithms could be applied to remove the soil moisture effects from spectra [18]. Wu et al. [19] identified a range of wavelengths where the first derivative of the reflectance spectra seems independent of the moisture content of the soil samples. They suggested only using these selected wavelength intervals to obtain moisture-independent estimates of SOC under field conditions. Minasny et al. [20] suggested that including a wide range of water contents in the calibration set could take care of the issue of moisture variation.

Multivariate models are commonly used to correlate data extracted from hyperspectral images to their corresponding chemical concentrations in soil samples [21]. Partial least squares regression (PLSR) is a useful model to predict dependent variables using a large number of independent variables. The advantage of PLSR compared with other multivariate models is the ability to manipulate large and noisy data sets [22,23]. Different soil hyperspectral data pretreatment methods and different modeling methods will affect the prediction accuracy of the model. Both principal components regression (PCR) and PLSR are linear chemometric tools used for the analysis of spectroscopic data for different applications. They are the most common modeling techniques for quantitative spectroscopy analyses in soils and have been extensively discussed in the literature [24,25,26]. They both represent techniques that are based on the decomposition of the spectral data into features that capture most of the variance that exists in the raw visible and near-infrared spectroscopic (VIS-NIRS) data and the creation of linear models using the scores of the most correlated features [27].

Previous studies mainly focused on the quantitative relationship between soil properties and hyperspectral reflectance in the laboratory, but using a handheld hyperspectral camera in solar light with different soil moisture levels is rarely reported. The objectives of this study are, therefore, (1) to evaluate whether the spectral data acquired via a portable hyperspectral camera outdoors could be used to predict SOM, AN, AP and AK in black soils; (2) to establish PLSR models between four soil properties and spectral data and compare the performances at different SM levels; and (3) to propose a series of feature wavelengths for the prediction of different soil properties.

## 2. Materials and Methods

### 2.1. Site Description, Soil Sample Collection and Preparation

The soil samples were collected in 2019 from an experimental site planted with maize in Harbin (45°40′ N, 126°37′ E), Heilongjiang Province of China. The soils involved in this work belong to black soil (classified as Mollisols in the U.S. taxonomy system). The samples were collected at depths of 0–20 cm. In total, 78 samples were used for this study. The soil samples were air-dried and sieved using a 2 mm sieve. Each sieved soil sample was divided into 4 sub-samples, among which one was air-dried, and the soil moisture was tested. Others were adjusted to different gravimetric water content levels around 20%, 30% and 40% before the hyperspectral scan. The water added ($W_{add}$) to adjust the soil moisture was calculated based on the following equations:

$$\frac{M_{soil}\times C_{original}+W_{add}}{M_{soil}+W_{add}}=C_{adjusted}$$

$$W_{add}=\frac{M_{soil}\times C_{adjusted}-M_{soil}\times C_{original}}{\left(1-C_{adjusted}\right)}$$

where $M_{soil}$ (20 g) is the weight of air-dried soil samples, $C_{original}$ (ranged from 2.2% to 3.5%) is the water content of air-dried soils, $W_{add}$ is the weight of water added and $C_{adjusted}$ is the aimed soil moisture (20%, 30% or 40%).

The soil moisture was adjusted in the Petri dishes, then covered with lids to prevent evaporation and kept for more than 18 h to make sure the water diffused well before scanning. In summary, 312 (78 × 4) sub-samples were used for hyperspectral data collection and modeling.

### 2.2. Soil Property Measurement

Laboratory analyses of SOM, AN, AP and AK were carried out by the Testing Center of Heilongjiang Academy of Black Soil Conservation and Utilization. Soil organic C was measured using an Elemental analyzer (VarioEL III, Elementar, Langenselbold, Germany). Soil available nitrogen (AN) was detected using an alkaline hydrolysis diffusion method [28]. Available phosphorus (AP) was measured by the Olsen method [29]. Available potassium (AK) was quantified using inductively coupled plasma-atomic emission spectrometry (ICPS-7500, Shimadzu, Kyoto, Japan). The ranges of soil properties are listed in Table 1.

### 2.3. Characteristics of the Hyperspectral Imaging System and Image Acquisition

**Figure 1:**
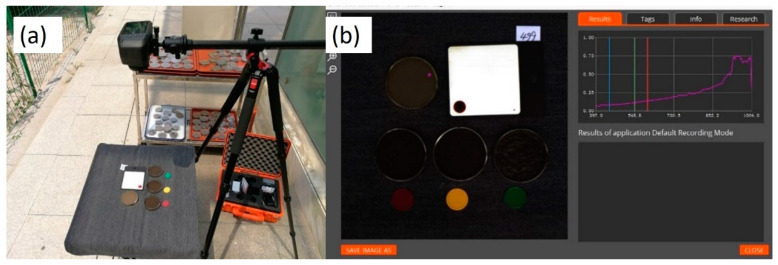
Hyperspectral image capturing outdoors (**a**) and data checking by Specim IQ Studio (Version 1.0) (**b**).

The hyperspectral images of soil samples were captured by a Specim IQ (Oulu, Finland) camera system. The measurements were performed based on the line scanner, i.e., push broom principle, and comprise the wavelength range of 400–1000 nm. Its spatial sampling, i.e., the number of pixels per line, is 512, and the spectral resolution is 7 nm, with 204 spectral bands across the wavelength range [30]. The spectral images were taken outdoors between 10:00 a.m. and 14:00 p.m. on sunny days. All the soil samples were scanned in the Petri dishes without disturbance, and the lids were removed before scanning. A white panel was used in each scanning as a reference target (Figure 1a). 

### 2.4. Spectral Data Extraction and Preprocessing

Hyperspectral data were extracted from the camera system (Figure 1b) and imported into a Scyven 1.3.0 (Scyllarus team, Canberra, Australia) to trace individual soil samples and resolve the regions of interest manually. The obtained data were first log transformed, then centered and scaled to unit variance. Principle component analysis (PCA) was performed on the processed data first to identify outliers and observe general trends. The PCA and partial least squares discriminant analysis (PLS-DA) were performed with the MetaboAnalyst web platform (Version 6.0).

### 2.5. Model Training and Validation

Partial least squares regression (PLSR) was used to fit the model between soil properties (SOM, AN, AP and AK) and hyperspectral data. The soil properties were continuous variable Y, and hyperspectral reflections were used as observable variable X. Cross-validation (10-fold) was used to determine the optimal number of PLS components. Multivariate analysis, including model training and validation, was performed with the R environment (version: 4.3.1) using the packages “pls”, “dplyr” and “MASS”. The assessment statistics used in the cross-validation were the coefficient of determination (R^2^), the root mean square error (RMSE) and the predicted variation (Q^2^):

$$R^{2}=1-\frac{SSE}{{SS}_{yy}}$$

$$SSE=\sum_{i=1}^{n}{\left(y_{i}-\hat{y}\right)}^{2}$$

$${SS}_{yy}=\sum_{i=1}^{n}{\left(y_{i}-\bar{y}\right)}^{2}$$

$$RMSE=\sqrt{\frac{1}{N}\sum_{i=1}^{N}{\left({\hat{y}}_{i}-y_{i}\right)}^{2}}$$

$$PRESS=\sum_{i=1}^{m}{\left(y_{i}-\hat{y}\right)}^{2}$$

$$SSY=\sum_{i=1}^{m}{\left(y_{i}-\bar{y}\right)}^{2}$$

$$Q^{2}=1-\frac{PRESS}{SSY}$$

where ${\hat{y}}_{i}$ is the predicted value, $y_{i}$ is the observed value and N is the number of data points. These statistics were used to quantify the accuracy of the soil property predictions.

## 3. Results and Discussion

### 3.1. Principal Component Analysis (PCA)

**Figure 2:**
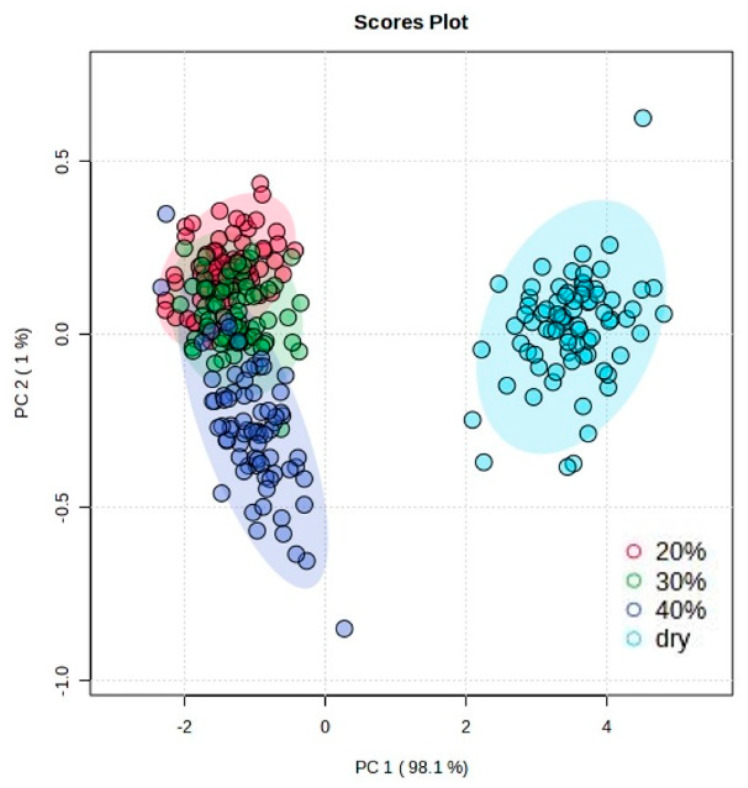
The profile of the spectral data separated into groups with different soil moisture content under principal component analysis (PCA).

In the current study, hyperspectral spectra were obtained in the wavelength range of 400–1000 nm, and 202 features were aligned for each measurement. Principal component analysis showed a general separation between the dry group and the other three groups with different moisture levels (20%, 30% and 40%). In multivariate analysis, PCA was performed on the processed data first to identify outliers and observe general trends [31]. Herein, PCA was applied to explore the overview of the spectrum profiles among the four groups. A clear separation between the dry group and the three re-watered groups is shown in Figure 2. The samples with 20%, 30% and 40% water content could also be distinguished, but less significantly compared to the dry samples. The results indicate that the soil samples with different moisture content could be profiled across the wavelength range of 400–1000 nm. This is in line with the findings of Liu et al. [32], who also demonstrated that VIS-NIR at 400–980 nm can successfully predict soil moisture content.

### 3.2. Performance of the PLS-DA Model

**Figure 3:**
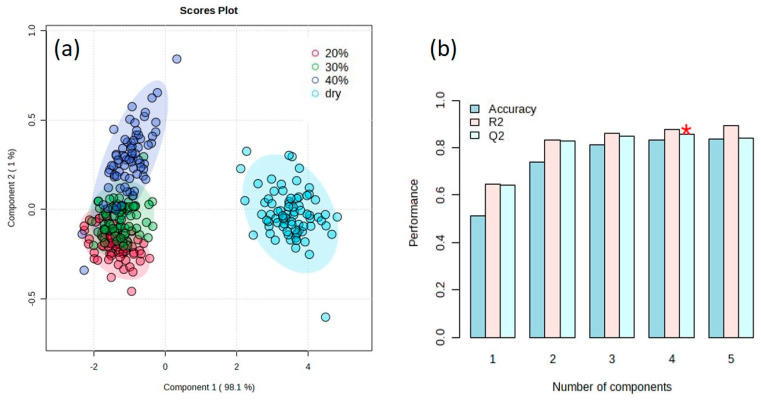
Profile of separated spectral data based on partial least squares discriminant analysis (PLS-DA) (**a**) and the accuracy, goodness of fit (R^2^) and predictive ability (Q^2^) of the PLS-DA model as assessed by 10-fold cross-validation (**b**). The red asterisk indicates the highest value of Q^2^.

**Figure 4:**
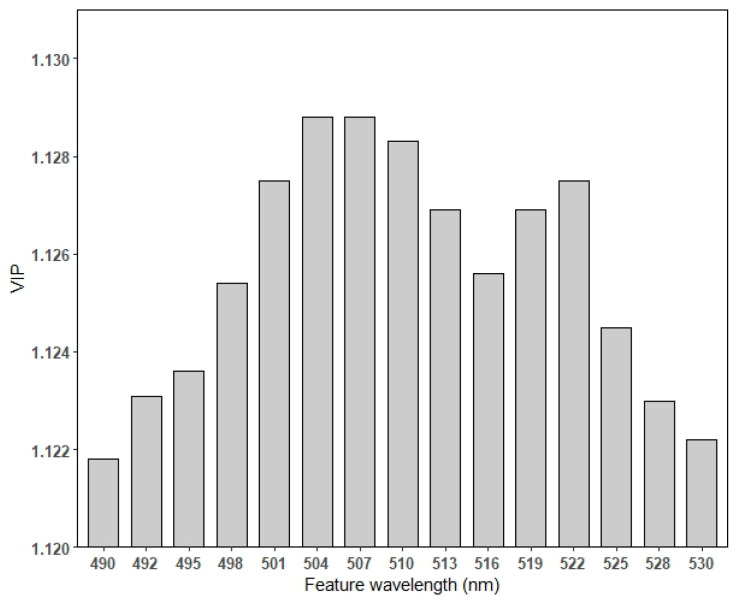
Top 15 feature wavelengths with relatively higher variable importance in projection (VIP) scores of PLS-DA.

The results of PLS-DA (Figure 3) confirmed that the hyperspectral data could be discriminated between the groups of different SMs with a discriminant power of Q^2^ = 0.85 (*p*-value = 2.0 × 10^−4^ obtained using a 10-fold crossed validation). A value of Q^2^ = 1 indicates a perfect discrimination. We present the top 15 feature wavelengths with relatively higher variable importance in projection (VIP) score, covering the wavelengths from 490 nm to 530 nm (Figure 4). The highest VIP score was 1.1288, which was achieved by the variable at 508 nm. Using machine learning to predict soil total nitrogen, organic carbon and moisture content, Morellos et al. [27] found that more wavelengths were highlighted as providing suitable prediction of SM, including 616, 684 and 823 nm. The effects of soil moisture on the prediction of SOC and other soil properties were further discussed in this study.

### 3.3. The Prediction of Soil Organic Matter, Available N, Available P and Available K

**Figure 5:**
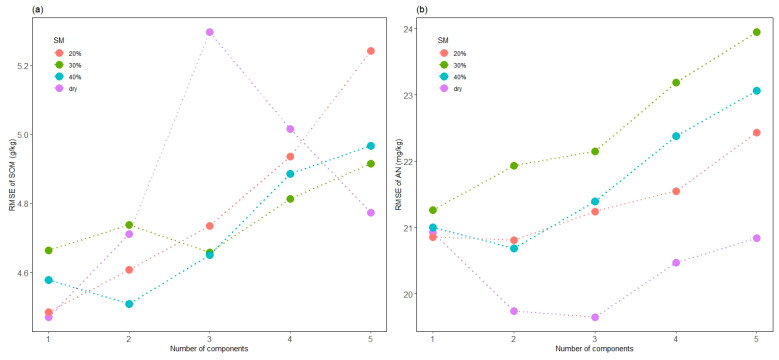
The performance of soil samples with different humidities to predict SOM (**a**) and AN (**b**) based on partial least squares models with 1 to 5 components.

PLSR models with one to five components were used to test the best performance model for predicting SOM, AN, AP and AK. In SOM prediction, the model with one component showed the best performance (RMSE = 4.47 g/kg, R^2^ = 0.4131) based on the dry samples (Figure 5a). The model based on samples of 30% SM, which is close to real soil moisture levels after irrigation in the field, showed a robust performance with the smallest RMSEP fluctuation and achieved a suitable performance with three components. Due to the presence of functional groups such as C-H, -COOH, -OH and N-H in soil organic compounds corresponding to spectral response at different wavelengths, spectral characteristics could be explained by different component numbers [9]. Thus, a model with more components is expected to describe more SOM features, improving the accuracy. However, our study achieved a contrary result, and accuracy decreased with the increased number of components, except for a fluctuation in the model based on dry samples.

In the prediction of AN, the model based on the air-dried soil samples with three components achieved the best performance (RMSE = 20.92 mg/kg, R^2^ = 0.4554), as shown in Figure 5b. The models with 20%, 30% and 40% SM showed rising RMSEP with an increasing number of components. Liu et al. [33] found that soil moisture significantly interfered with the correlation between reflectance and soil nitrogen content. The RMSE variations of the models based on different SMs follow a similar trend but could be distinguished by SM. This suggests that the predictive abilities of models were gradually improved as the soil moisture decreased.

According to the classification of [34], soil nitrogen and organic matter are primary properties and show direct spectral responses in the NIR region, whereas soil phosphorus and potassium are secondary properties that do not possess direct spectral responses for NIR predictions. They are predictable because of their correlation with certain primary properties. However, because of the complexity of overtones and combinations in the NIR region [35], it is difficult to explain what fractions of soil phosphorus and potassium are correlated with NIR spectra from 400 to 1000 nm [36]. 

**Figure 6:**
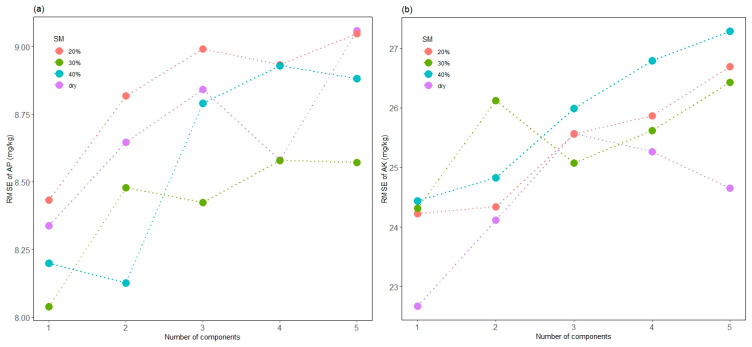
The performance of soil samples with different humidities to predict AP (**a**) and AK (**b**) based on partial least squares models with 1 to 5 components.

In the prediction of AP and AK, we can see the model accuracies fluctuate and deteriorate with increasing components. AP was found to be measurable with different degrees. The AP model of 30% SM with one component showed the best performance in this study (RMSE = 8.04 mg/kg, R^2^ = 0.4360), followed by 40% SM with two components (Figure 6a). This might partly be attributed to the covariation of moisture content. Maleki et al. [37] suggested that the indirect correlation between P and soil spectra could be attributed to the water. P fraction in the water phase of the soil correlates well with the spectral signal, and more water implies more P. If this is the case, it would explain that P can be better measured in fresh soil samples, as found by [38].

The model for air-dried samples with one component outperformed others in the prediction of AK (RMSE = 22.67 mg/kg, R^2^ = 0.5498) (Figure 6b). The suitable prediction of AK in this work may be attributed to the correlation with illite, which is the major component of clay minerals that can be directly predicted by spectra data [36]. Drake [39] studied the spectral response of evaporite minerals and found that the vast majority of evaporite minerals had diagnostic spectra due to the vibration of H_2_O, NH_4_ and NO_3_ bonds. Generally, explaining the correlation between spectrally inactive properties and visible spectra is still difficult; improving the prediction accuracy for P and K should be further investigated.

### 3.4. Feature Wavelengths for PLSR Modeling

**Figure 7:**
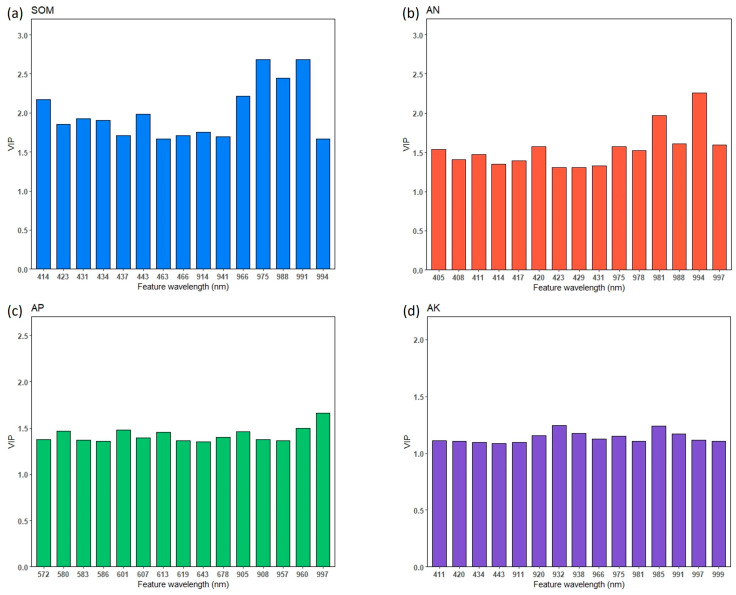
Top 15 feature wavelengths used in PLSR prediction for SOM (**a**), AN (**b**), AP (**c**) and AK (**d**).

The variable importance in the projection (VIP) was used to identify the important wavelengths used in PLSR predictions. If the VIP value for a specific wavelength is greater than 1, this spectral wavelength is then considered to be important [33,40]. We list the top 15 wavelengths for each soil property prediction in Figure 7a. Bands near 414, 423, 431, 443 and 914–991 nm were identified as significant for SOM predictions. This result agrees with reports by [23], who used two portable hyperspectral sensor-based instruments to predict key soil properties in Canadian soils and reserved 911 and 986 nm for predicting soil organic carbon. In this study, wavebands near 405, 420, 431 and 975–994 nm are most useful for the prediction of AN (Figure 7b). Yang et al. [9] found that sensitive bands of soil nitrogen in the VIS range were densely distributed in the region of 400–440 nm, which was consistent with our findings. Vohland et al. [41] also identified a series of similar key wavelengths for carbon fractions and nitrogen in the 450–675 nm region. 

The wavelengths selected for AK were mainly distributed in the region of 920–997 nm (Figure 7d), which is similar to the findings of Guo et al. [42]. The reflectance features of AK and AP rely on indirect inversion of other soil component contents because there are no direct responses associated with them in the VIS-NIR wavelength range; the element types are complex and inconsistent [37], and they usually exist at low concentrations [35,36]. As shown in Figure 7c, the most useful wavelengths associated with AP prediction are 572–586 nm, 601–619 nm, 643–678 nm and 905–997 nm. As in the experiments of [38], the reflectance of the P components was found to be different if measured on dry or wet soils, which was also supported by the study of Maleki et al. [37]. These results might be useful in developing feature band fixed spectrometers in different application scenarios.

### 3.5. Effects of Water Content on Prediction

The presence of water generally reduces the VIS-NIR reflectance [16]. Wetting induces a darkening of the soil and smooths the spectra, particularly brightness in the shortwave VIS region. As shown in Figure 5, the prediction of AN, AK and SOM deteriorated significantly with increasing soil moisture. However, as the SM decreases, the prediction accuracy improves gradually. Moisture can mask these peaks due to other OH bands present; it can also cause spectral differences due to interactions between water and other components [43]. When soil moisture increases, soil particles adsorb the water, and then micro- and macropores are filled with water. For VIS wavelengths, this causes a change in the relative refractivity at the soil particle surface, while there is little effect on reflectance when water covers micro- and macropores. Longer wavelengths are able to strongly adsorb water, producing a significant change in reflectance even when the moisture content is at field capacity [13].

The variation in soil moisture has been reported to cause poorer calibration properties [20,44]. However, Mouazen et al. [38] demonstrated that phosphorus is better predicted in wet samples than in dried samples, which is consistent with the current study. The model for predicting AP at 30% SM with one component showed the best performance in prediction. This is in contrast with the prediction of all other elements, where the best prediction was found on dried soil samples [37]. 

## 4. Conclusions

Soil samples with different moisture levels could be discriminated by the PLS-DA model based on spectral data in the wavelength range of 490–530 nm. SOM, AN and AK could be well predicted by PLSR models for air-dried samples in this study. AP could be better predicted by the model for 30% soil moisture. Wavelengths of 414, 423, 431, 988 and 994 nm are important for both SOM and AN prediction. The sensitive wavelengths for AP were distributed in the regions of 572–678 nm and 905–997 nm and for AK in the regions of 411–443 nm and 911–1000 nm. Considering the imaging time of spectrometers, selected wavelengths may be more effective than using a full-range spectrum for quantitative modeling. Spectrometers focused on selected feature bands have the advantage of saving time. The feature bands proposed in this study could be employed to develop wavelength fixed portable spectrometers for outdoor use, which will be our next step in the work related to on-site soil monitoring and modeling.

## Figures and Tables

**Table 1:** Basic information of soil samples.

Soil Depth cm	pH	SOM g/kg	Available N mg/kg	Available P mg/kg	Available K mg/kg
0–20	6.6–7.1	21.42–45.56	114.23–233.23	11.96–45.73	77.25–164.52

## Data Availability

Data are contained within the article.
